# Integrating transposable elements into blood gene regulatory networks reveals connectivity loss for schizophrenia genes

**DOI:** 10.1172/JCI203491

**Published:** 2026-09-01

**Authors:** Helena Reyes-Gopar, Matthew L. Bendall, Rodrigo R.R. Duarte, Timothy R. Powell, Douglas F. Nixon

**Affiliations:** 1 Northwell Health, Feinstein Institutes for Medical Research, Manhasset, New York, USA.; 2 Department of Biostatistics and Bioinformatics, Computational Biology Institute, Milken Institute School of Public Health, The George Washington University, Washington, DC, USA.; 3 Social, Genetic & Developmental Psychiatry Centre, Institute of Psychiatry, Psychology & Neuroscience, and; 4 Department of Medical & Molecular Genetics, Faculty of Life Sciences & Medicine, King’s College London, London, United Kingdom.

**Keywords:** Genetics, Neuroscience, Bioinformatics, Biomarkers, Schizophrenia

## Abstract

In patients with schizophrenia we found that genes and dark genome disconnect. This opens new areas for possible diagnostics and treatments.

**To the Editor:** Studies of schizophrenia (SCZ) disease etiology support neuro- and immunobiological hypotheses, and high heritability in twin studies suggests a genetic component (1, 2). We recently showed human endogenous retroviruses (HERVs), a class of transposable elements (TEs), are associated with SCZ risk in the adult brain (3). As TEs play important roles in immune gene regulation (4), we used a systems biology approach to characterize TE-gene regulatory networks in PBMCs from treatment-naive patients with first-episode SCZ (5). We analyzed publicly available RNA-seq data from 84 patients with SCZ and 97 individuals treated as healthy controls (HCs) (5), quantifying 21,622 canonical genes (CGs), 8,438 long interspersed nuclear elements-1 (L1s), and 4,275 HERVs from PBMCs (Supplemental Methods; supplemental material available online with this article; https://doi.org/10.1172/JCI203491DS1).

Mutual-information networks have been applied in cancer research (6) (Supplemental Methods), but not in psychiatric disorders. We constructed gene and TE mutual-information networks using identical node sets: 15,822 CGs and 219 intergenic TEs (Supplemental Methods). We analyzed intergenic TEs to avoid confounding coexpression from overlapping CG transcription. Specific L1 and HERV loci were part of the HC network with high mutual-information edges to CGs (Figure 1, A–C, and Supplemental Figure 2B). The SCZ network exhibited structural disruption: 1,725,564 edges compared with 3,538,017 in the HC network, with decreased density from 0.0275 to 0.0134, and reduced clustering coefficients in the SCZ network (0.188 HC, 0.139 SCZ) (Supplemental Figure 1A). Both HERV and L1 nodes had significantly higher median degrees than CGs in HC and SCZ networks (bootstrap test, 100,000 iterations; *P* < 0.01 for all comparisons) (Figure 1D).

TEs functioned as highly connected nodes in both networks (Figure 1, A and B). Overrepresentation analysis (1-sided Fisher’s exact test, Benjamini-Hochberg correction) of the top 5% most TE-connected CGs revealed an asymmetry between conditions. In HCs, only 1 pathway reached significance (regulation of organ morphogenesis, L1 elements; adjusted *P* ≤ 0.005). In SCZ, CGs coexpressed with L1s, and HERVs were enriched in 15 and 17 pathways, with 12 shared between TE classes (adjusted *P* < 0.05; Supplemental Figure 3), converging on complement activation, Fc receptor signaling, and phagocytosis. HERV-connected genes were additionally enriched for antimicrobial humoral response, indicating that TE-gene coexpression rewiring in SCZ preferentially involves innate immune and complement pathway genes, consistent with an immune component of SCZ.

Genes previously reported to be involved in SCZ (e.g., *NRG1*, *SETD1A*) had substantial changes in the number of edges (Figure 1, A and B, and Supplemental Figure 1A). TEs coexpressed with SCZ genes in the HC network presented a complete rewiring of their edges in SCZ, specifically *L1FLnI_2p13.1f*, *HERV30_4q13.2c*, *HERVL_4q32.1a*, *L1FLnI_8q13.3e*, and *HERVFH21_16p11.2* (Supplemental Figure 4C). These data demonstrate that TEs constitute integral components of gene regulatory networks in PBMCs and undergo systematic dysregulation in first-episode SCZ.

Patients with SCZ showed widespread transcriptional dysregulation, with 11,531 CGs, 1,745 L1s, and 989 HERVs differentially expressed (adjusted *P* < 0.01; Figure 1E). The largest expression changes were dominated by TEs rather than CGs, with L1s and HERVs comprising 80.5% of features showing ≥ 2.83-fold changes and exhibiting near universal downregulation (Figure 1E and Supplemental Figure 1, C and D). We discovered that the expression pattern of the top 50 most dysregulated features, including 39 CGs, 10 L1s, and 1 HERV, distinguished SCZ from HC (Figure 1F). Among CGs, the connexin genes GJB2/GJB6, which are typically associated with hearing functions in cochlear tissue, showed the highest upregulation in PBMCs in patients with SCZ (Supplemental Figure 1G). Immune pathway enrichment among TE-connected genes should be interpreted with caution. Mechanistically, the downregulation of TEs in SCZ, combined with loss of TE-gene network connectivity, suggests a model in which TE silencing disrupts coregulatory relationships with immune and neurodevelopmental genes. Several genes connected to TEs in these networks, including NRG1 and SETD1A, have established roles in immune cell biology beyond their canonical functions in neurobiology, consistent with their coexpression with TEs in blood. However, while blood represents an accessible tissue for biomarker discovery, the extent to which these CG and TE network alterations parallel CNS mechanisms remains unknown.

Current diagnostic and treatment options for SCZ are not based on molecular data and overlook the immune component. The therapeutic and diagnostic potential of the inferred regulatory interactions between genes and TEs in peripheral blood of patients with SCZ warrants further investigation. For detailed methods, information regarding sex as a biological variable, statistics, study approval, author contributions, data availability, and acknowledgments, see the supplemental materials.

## Conflict of interest

The authors have declared that no conflict of interest exists.

## Funding support

Psychiatry Research Trust grant (to RRRD and TRP).
Medical Research Council (UK Research and Innovation) New Investigator Research Grant (MR/W028018/1 to TRP).
National Institute for Health and Care Research Biomedical Research Centre: Maudsley at South London and Maudsley NHS Foundation Trust and King’s College London (TRP).

## Supplementary Material

Supplemental data

Supporting data values

## Figures and Tables

**Figure 1:**
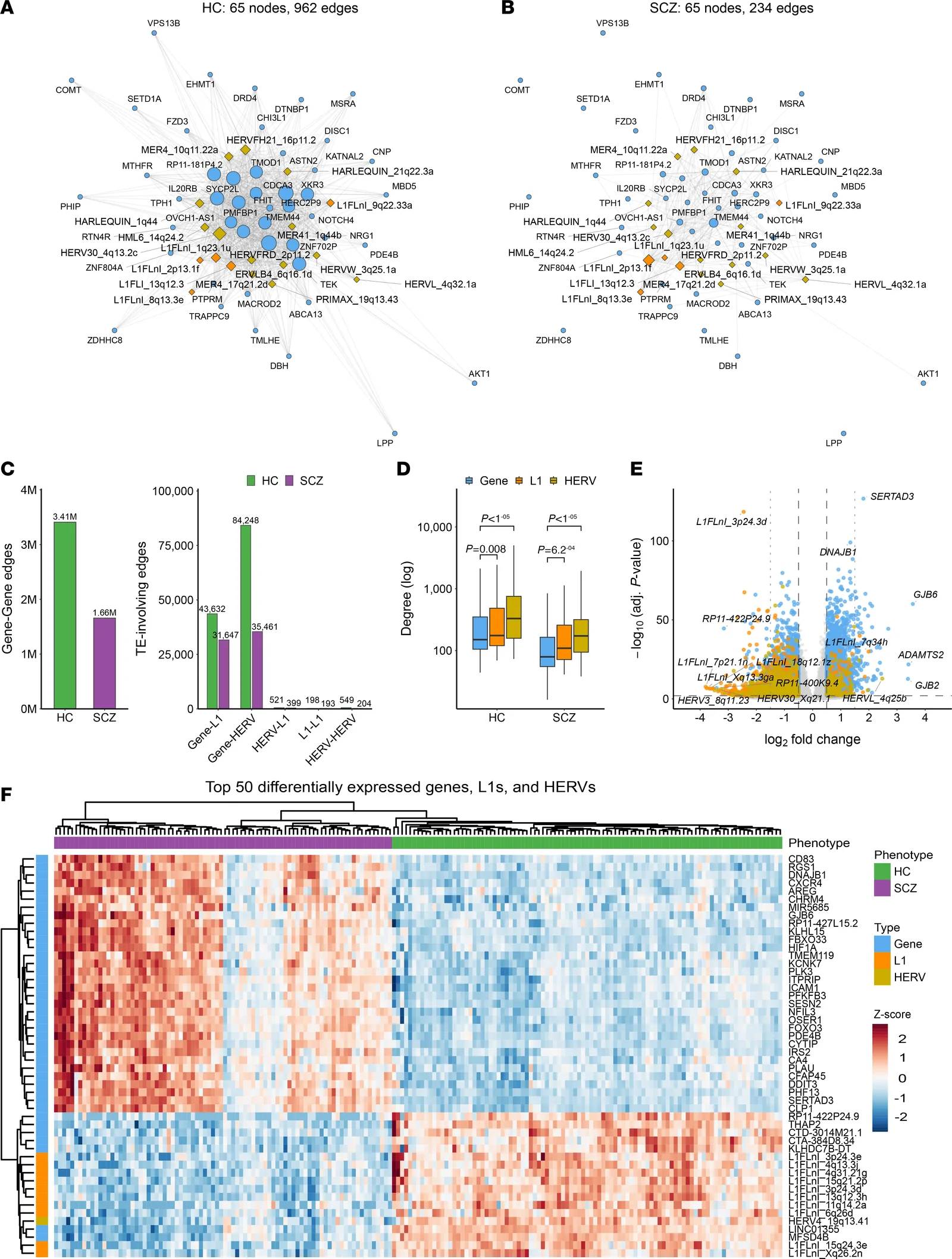
Mutual-information networks and differentially expressed genes differentiate PBMCs of SCZ and HC networks. (**A** and **B**) Mutual-information (MI) coexpression networks for HC (**A**; 962 edges) and SCZ (**B**; 234 edges) groups, showing 65 high-degree nodes selected from networks (HC: 16,041 nodes, 3.54 million edges; SCZ: 16,041 nodes, 1.73 million edges). Circles represent CGs (blue); diamonds represent TEs (orange for L1s, gold for HERVs). Node size is proportional to degree in the network. (**C**) Bar plots of genome-wide MI edge counts by transcript pair type for HCs (green) and SCZ (purple). (**D**) Box-and-whisker plots of node degree for genes, L1s, and HERVs in the HC and SCZ networks. Boxes span 25th–75th percentiles; center lines indicate medians; whiskers extend to 1.5× the interquartile range. (**E**) Volcano plot of differential expression (SCZ vs. HC) showing log_2_ fold change (FC) versus –log_10_ adjusted *P* value for genes (blue), L1 elements (orange), and HERVs (gold). Dashed lines indicate thresholds of adjusted *P* < 0.01 and |log_2_FC| > 0.5. (**F**) Heatmap of the top 50 upregulated and downregulated transcripts ranked by combined score (–log_10_[adjusted *P*] × |log_2_FC|). Color scale represents row-wise *z* scores of variance-stabilized transformed counts. Differential expression was assessed using DESeq2 with correction. Node degree differences (**D**) were tested by nonparametric bootstrap (100,000 iterations).

## Version 1 09/01/2026

Electronic publication
