# Supplementary material for: Integrating transposable elements into blood gene regulatory networks reveals connectivity loss for schizophrenia genes
Source: J Clin Invest. 2026 Sep 1;136(17):e203491. doi: 10.1172/JCI203491 (PMC13528912; doi:10.1172/JCI203491)
Supplement: Supplementary file 1 [file jci-136-203491-s145.pdf]

Integrating transposable elements into blood gene regulatory networks reveals connectivity loss for schizophrenia genes

Helena Reyes-Gopar[1*], Matthew L. Bendall[2], Rodrigo R.R. Duarte[3,4], Timothy R. Powell[3], Douglas F. Nixon[1#]

[1]Northwell Health, Feinstein Institutes for Medical Research, Institute of Translational Research, Manhasset, NY, 11030, USA

[2]Department of Biostatistics and Bioinformatics, Computational Biology Institute, Milken Institute School of Public Health, George Washington University, Washington, DC 20052, USA

[3]Social, Genetic & Developmental Psychiatry Centre, Institute of Psychiatry, Psychology & Neuroscience, King's College London, London, United Kingdom

[4]Department of Medical & Molecular Genetics, Faculty of Life Sciences & Medicine, King's College London, London, United Kingdom.

[#]Corresponding Author: Douglas F. Nixon, MD, PhD, Feinstein Institutes for Medical Research, 350 Community Drive, Institute of Translational Research, Manhasset, NY, 11030, USA

Tel +1 516 684 0747

Email: dnixon1@northwell.edu

[*]Lead contact. E-mail: hreyesgopar@northwell.edu

The authors have declared that no conflict of interest exists.

Helena Reyes-Gopar et al.

Table of Contents

Supplementary methods

Supplementary figure legends

Supplementary figures

Supplementary methods:

Sex as a biological variable

Both male and female sexes were involved in this study. The SCZ and HC groups differ in sex composition and mean age, reflecting the composition of the publicly available dataset (Pan et al., GEO: GSE243841). Individual-level sex data were not deposited in GEO by the original investigators; only aggregate demographics are available. Consequently, sex was not considered as a biological variable in the differential expression model or mutual information network inference.

RNA-Seq data collection

Bulk RNA-seq data from 181 samples were obtained from the study by Pan et al (1):

Pan B, Li X, Weng J, Xu X et al. Identifying periphery biomarkers of first-episode drug-naïve patients with schizophrenia using machine-learning-based strategies. *Prog Neuropsychopharmacol Biol Psychiatry* 2025 Mar 20;137:111302. PMID: 40015618

Participant Characteristics:

Schizophrenia group (n=84): First-episode, drug-naïve patients with schizophrenia.

Control group (n=97): Healthy controls, matched for demographic characteristics.

All participants were recruited following appropriate ethical approval and informed consent procedures as described in the original study. As this is a reanalysis of publicly available data, we are limited to the clinical and demographic information provided by the original investigators.

Reference Genome Assembly

All RNA-seq data were aligned to the human reference genome GRCh38.d1.vd1, ensuring compatibility with current genomic standards.

Canonical Genes Annotation

Canonical gene annotation was performed using GENCODE version 38, which provides 60,605 gene models that include canonical protein-coding genes (2).

Transposable Element Classification

Transposable Elements (TEs) comprise approximately 45% of the human genome and are classified into two major classes based on their mechanism of transposition. Class I elements (retrotransposons or retroelements) mobilize via an RNA intermediate through a "copy-and-paste" mechanism and include long interspersed nuclear elements (LINEs, of which LINE-1/L1 is the only family capable of autonomous retrotransposition), short interspersed nuclear elements (SINEs, including Alu elements), and endogenous retroviruses (HERVs/LTR retrotransposons). Class II elements (DNA transposons) mobilize via a DNA intermediate through a "cut-and-paste" mechanism (3).

In this study we focus on full-length L1 elements and HERV proviruses. Both classes are transcribed by RNA polymerase II from their own internal or LTR-derived HERV promoters, producing autonomous transcripts that are distinct from read-through transcription of neighboring canonical genes. HERVs are genomically fixed, no active HERV retrotransposition has been documented in modern humans. The regulatory relevance of HERVs in this study therefore derives from their transcriptional activity and co-expression relationships with canonical genes.

SINEs were excluded because they are transcribed by RNA polymerase III and are therefore subject to different regulatory genomics processes. DNA transposons are 2-3% of the human genome and lack autonomous transcriptional activity.

Transposable Elements (TEs) Annotation

TE quantification utilized the retro.hg38.v1.gtf annotation, a curated database containing 13,545 LINE1 elements and 14,968 HERV proviruses. This specialized annotation addresses limitations in standard repeat databases by defining HERV transcriptional units through systematic combination of RepeatMasker annotations from identical subfamilies located in adjacent genomic regions. Unlike conventional approaches that treat internal protein-coding regions and flanking LTRs as separate annotations, this methodology preserves the functional organization of complete HERV proviral units. The annotation database and supporting documentation are publicly available at https://github.com/mlbendall/telescope_annotation_db

RNA-seq Alignment

RNA-seq reads were aligned to the human reference genome using STAR aligner (4) version 2.7.10b with parameters optimized to preserve multi-mapped reads (outFilterMultimapNmax: 500, winAnchorMultimapNmax: 500, outFilterMultimapScoreRange: 5) essential for transposable element analysis.

TE Quantification

Transposable element transcript quantification was performed using Telescope (5), a computational tool specifically designed to resolve multi-mapped reads corresponding to transcripts that originate from repetitive genomic sequences. Telescope operates at the individual fragment level, enabling locus-specific quantification of individual TE insertions with precise genomic coordinates.

Telescope quantifies expression by modeling the probability that each multi-mapped read fragment originates from each potential TE locus in the annotation. An expectation-maximization (EM) algorithm optimizes parameters representing relative expression levels (π) and alignment quality distributions (θ) across all dataset fragments until convergence.

Telescope was applied to each sample's aligned BAM file using the following parameters: "--theta_prior 200000 --max_iter 1000". The theta prior of 200,000 provided regularization equivalent to adding 200,000 non-unique reads to stabilize parameter estimation, while the maximum iteration limit of 1,000 ensured algorithm convergence. Final transcript counts for HERV proviruses and Long Interspersed Nuclear Elements (LINE1/L1) were extracted from the Telescope report using the "exclude" reassignment mode.

In the Telescope 'exclude' reassignment mode, fragments with multiple equally probable best assignments are excluded from final counts. This strategy was selected to maximize quantification confidence and minimize false-positive TE expression signals. While this approach potentially underestimates true TE expression levels by discarding some ambiguous fragments, it ensures that reported counts reflect high-confidence locus-specific assignments, which is particularly important when downstream analyses depend on accurate co-expression estimation across samples. Alternative reassignment modes (e.g., 'average' or 'all') that distribute or retain ambiguous fragments were not

used, as they could introduce correlated noise across TE loci and inflate co-expression estimates in the mutual information networks.

Mutual Information Network Construction

Mutual Information (MI) Gene Regulatory Networks (GRNs) are models that can map the regulatory relationships among genes. The inference of these networks from high throughput data like RNA-seq has become a central discipline within computational and systems biology. GRNs provide a powerful mechanistic link between genotype and cellular phenotype, explaining how coordinated gene expression programs orchestrate complex biological processes. To date, they have mostly been used to study dysregulation in cancers (6, 7), and this is the first study to our knowledge to use MI GRNs including TEs in the context of a psychiatric disorder. Schizophrenia is a highly polygenic disorder, with hundreds of common variants of small individual effects contributing to genetic risk. Network based approaches offer a framework to understand how coordinated dysregulation across multiple genes may contribute to disease phenotypes, complementing single-gene analyses.

This approach enabled locus-specific quantification of transcripts from individual TE loci in the PBMC's bulk RNA-seq data. Across the full annotation detection rates showed a similar bimodal distribution for all three feature types: most loci were either detected in nearly all samples or in very few, with relatively few loci at intermediate detection rates (Supplementary Figure 1B). This pattern yields robust signal for downstream coexpression and differential expression analyses.

Gene Regulatory Network Construction

Gene regulatory networks were constructed using the ARACNe (Algorithm for the Reconstruction of Accurate Cellular Networks) approach implemented in the minet R package (8). Prior to network

construction, expression data underwent stringent filtering to ensure robust network inference. Canonical genes were retained if they had ≥10 counts in ≥50% of samples and mean expression ≥10 counts, while transposable elements were retained if they had ≥10 counts in ≥50% of samples and mean expression ≥5 counts due to their typically lower expression levels. These filtering criteria were applied independently of the differential expression analysis filters to select features with sufficient expression breadth and depth for reliable mutual information estimation. The final feature set comprised 15,822 canonical genes and 1,350 transposable elements (811 L1s and 539 HERVs), which were further refined to 219 intergenic TEs following genomic context filtering (see TE Genomic Context Filtering below). Expression data were normalized using variance-stabilizing transformation (VST) from DESeq2.

Mutual information matrices were calculated separately for schizophrenia (n=84) and healthy control (n=97) samples using the Spearman rank correlation estimator, which converts pairwise Spearman correlations (ρ) to mutual information values via the transformation $MI = -0.5 \times \log(1 - \rho^2)$. This estimator was chosen for its robustness and its ability to capture monotonic nonlinear dependencies. The resulting mutual information values are non-negative and unbounded, with higher values indicating stronger statistical dependency between feature pairs. The ARACNe algorithm was then applied with data processing inequality (DPI) tolerance $\varepsilon=0.05$. This DPI step iteratively examines every triplet of mutually connected nodes and removes the weakest edge, ensuring the resulting network is enriched for direct rather than transitive regulatory relationships.

Networks were constructed using identical feature sets (canonical genes, L1, and HERV transcripts) as nodes to enable direct topological comparisons between conditions. Edge weights in the final

networks represent mutual information values retained after ARACNe pruning. These values reflect the strength of pairwise co-expression dependencies but are not normalized to a fixed scale (Supplementary Figure 2A); accordingly, downstream analyses of network topology (e.g., node degree, edge counts) were based on edge presence rather than edge weight magnitude. The mutual information edges in these networks represent statistical co-expression dependencies and do not necessarily reflect direct physical regulatory interactions (Supplementary Figure 2B-C). However, the application of data processing inequality removes indirect associations, enriching for relationships more likely to reflect regulatory coordination.

TE Genomic Context Filtering

To avoid correlations arising from host canonical gene expression, transposable elements were filtered based on genomic context using a curated TE annotation database (TE_annotation.v2.0.tsv) available at https://github.com/liniguez/Telescope_MetaAnnotations.

Of the 1,350 TEs passing expression-based filtering, 219 (16.2%) were classified as intergenic, 501 (37.1%) as intronic, and 630 (46.7%) as exonic based on their genomic location relative to canonical genes. Only intergenic TEs were retained for network analysis, as these represent independent transcriptional units whose co-expression patterns cannot be attributed to transcriptional read-through from host genes. TEs located within or overlapping protein-coding genes (exonic and intronic) and TEs lacking genomic context annotation were removed, yielding a final network of 16,041 nodes: 15,822 canonical genes and 219 intergenic TEs (99 L1s and 120 HERVs).

To validate that this filtering did not alter our primary findings, we compared network metrics before and after TE genomic context filtering. The HC network retained 86.8% of its edges (3,538,017 of 4,077,079) and the SCZ network retained 85.8% (1,725,564 of 2,011,700) after filtering. All key

results were preserved: the HC network maintained greater overall connectivity than the SCZ network (average degree 441.1 vs 215.1, respectively), and intergenic TEs maintained higher average degree than canonical genes in both conditions (HC: 595.5 vs 439.0; SCZ: 313.7 vs 213.8).

Network Topology Analysis

Network properties were calculated using the igraph R package (9). Basic topological metrics included node count, edge count, network density (ratio of observed to possible edges), average node degree (mean number of connections per node), and global clustering coefficient (measure of network modularity).

Node degree (i.e., the number of edges per node) was calculated for each feature and stratified by biotype (Gene, LINE1, HERV) and phenotype (HC, SCZ) (Figure 1C). To assess whether TE loci exhibited significantly higher connectivity than canonical coding genes within each phenotype, we performed a non-parametric bootstrap test with 100,000 iterations. In each iteration, node degrees were resampled with replacement within each biotype, and the median degree difference (TE − Gene) was computed. A one-sided p-value was calculated as the proportion of bootstrap replicates in which the observed median TE degree did not exceed the median Gene degree. Confidence intervals (95%) were derived from the 2.5th and 97.5th percentiles of the bootstrap distribution. Both L1 and HERV loci exhibited significantly higher median node degree than protein-coding genes in both HC and SCZ networks (L1 vs Gene: $p = 0.008$ and $p = 6.2 \times 10^{-4}$; HERV vs Gene: $p < 1 \times 10^{-5}$ for both phenotypes), indicating that TE loci occupy highly connected positions within PBMCs co-expression networks regardless of disease status.

Hub nodes were defined as features in the top 10% of the degree distribution (Supplementary Figure 4A-B). Statistical significance of degree differences between feature types was assessed using

Wilcoxon rank-sum tests. Network disruption in schizophrenia was quantified by comparing topological metrics between conditions and calculating relative changes in connectivity.

TE Subnetwork Analysis

TE-specific subnetworks were extracted by creating induced subgraphs containing only transposable element nodes and their direct connections. Subnetwork properties including node count, edge count, density, clustering coefficient, and largest connected component size were calculated for both conditions.

Functional enrichment of TE-connected genes

To identify biological processes associated with TE-gene regulatory relationships, we performed over-representation analysis (ORA) on genes stratified by their connectivity to transposable elements in the HC and SCZ co-expression networks. For each network, we computed the TE-degree of every gene, defined as the number of TE nodes to which a gene is connected by a significant MI edge. TE-degree was calculated separately for L1 elements (n = 99 full-length L1HS/L1PA loci) and HERV elements (n = 120 loci from diverse HERV/LTR families). We defined "highly TE-connected" genes as those with TE-degree at or above the threshold corresponding to the top 5% of all genes in the network. This adaptive thresholding yielded foreground sets of comparable size across conditions: 869 genes for L1 in HC (degree $\geq$ 12), 800 for HERV in HC (degree $\geq$ 23), 952 for L1 in SCZ (degree $\geq$ 7), and 898 for HERV in SCZ (degree $\geq$ 9). The background universe comprised all 15,822 genes present in the network.

For each foreground gene set, we tested for pathway over-representation using one-sided Fisher's exact tests against four gene set collections from the Molecular Signatures Database (MSigDB v2024.1): Hallmark (50 gene sets), Reactome (1,787 pathways), KEGG Legacy (186 pathways), and Gene Ontology Biological Process (7,583 terms). Gene sets with fewer than 15 or more than 500 genes overlapping the network were excluded. P-values were adjusted for multiple testing using the Benjamini-Hochberg procedure within each combination of TE type, condition, and gene set collection. Pathways were classified as "conserved" (significant in both HC and SCZ, adjusted $p < 0.05$), "lost in SCZ" (significant in HC only), or "gained in SCZ" (significant in SCZ only) to identify condition-specific changes in TE-gene regulatory relationships (Supplementary Figure 3).

Pathway enrichment for immune-related processes among TE-connected genes should be interpreted with caution, as PBMCs are immune cells. The more informative finding is not the presence of immune pathways per se, but the differential organization of TE-immune gene co-expression between SCZ and HC. Whether this reflects a primary immunological perturbation, or a consequence of systemic factors cannot be determined from bulk data. Single-cell RNA-seq studies incorporating TE quantification in immune cell populations will be necessary to resolve this question and to distinguish compositional effects from within-cell regulatory changes.

Schizophrenia-Associated Gene Network Analysis

To investigate the network behavior of genes previously implicated in schizophrenia pathogenesis, we obtained a curated list of schizophrenia-associated genes from the MalaCards database (https://www.malacards.org/). This database aggregates gene-disease associations from multiple sources and utilizes a weighted scoring system for genes derived from high-confidence sources (e.g., OMIM and ClinVar). To prioritize high confidence associations, we selected 68 genes with a

MalaCards composite score >= 40. This threshold was chosen to include genes with established clinical and experimental evidence (such as genetic linkage), while excluding lower scoring associations derived solely from automated text mining. Of these, 36 genes were successfully mapped to our networks for further analysis.

For each schizophrenia-associated gene present in our networks, we calculated degree changes between healthy control and schizophrenia conditions to identify features showing significant connectivity alterations in disease. Degree changes were defined as the difference in node connectivity (number of edges) between conditions, with positive values indicating increased connectivity in schizophrenia and negative values indicating decreased connectivity.

TE-Schizophrenia Gene Interaction Rewiring Analysis

To examine the rewiring of regulatory relationships between transposable elements and schizophrenia genes, we identified all TEs directly connected to schizophrenia-associated genes in the healthy control network. For each TE-schizophrenia gene pair showing connectivity in controls, we assessed whether this connection was preserved, lost, or altered in the schizophrenia network.

Edge rewiring was quantified by comparing the presence/absence of specific TE-gene connections between conditions. TEs showing complete loss of connections to schizophrenia genes (present in HC network but absent in SCZ network) were prioritized as examples of disease-specific network disruption. This analysis identified specific transposable elements (*L1FLnI_2p13.1f, L1FLnI_8q13.3e, and HERVL_4q32.1a)* that lost regulatory relationships with schizophrenia-associated genes, suggesting their potential role in disease-related network reorganization (Supplementary Figure 4C).

Network Visualization

To visualize the co-expression networks, we extracted representative subnetworks from the full HC and SCZ graphs, which contain all 16,041 nodes (15,822 genes and 219 intergenic TEs) and their respective edges. Given the scale of the full networks (HC: 3,538,017 edges; SCZ: 1,725,564 edges), direct visualization of all nodes and edges is impractical. We therefore selected a biologically motivated subset of nodes for display purposes, while noting that any subset of nodes can be similarly extracted and visualized from the full networks.

The displayed subnetwork (Figure 1A) was constructed by selecting nodes from four categories: (1) three TEs of interest identified through differential expression and network analyses (*L1FLnI_2p13.1f, L1FLnI_8q13.3e, HERVL_4q32.1a*); (2) the top 15 TE hubs ranked by degree centrality in the HC network; (3) all schizophrenia-associated genes present in the network (n = 32), drawn from the curated list of 68 known SCZ genes; and (4) the top 15 non-SCZ gene hubs ranked by degree centrality in the HC network. This produced a subnetwork of 65 nodes.

Node positions were determined using the Fruchterman-Reingold force-directed layout algorithm applied to the HC subnetwork, and the same coordinates were used for the SCZ panel to enable direct visual comparison of connectivity changes between conditions. Node size was scaled proportionally to each node's degree in the full network (not the subnetwork) to preserve information about global network importance. All network analyses and visualizations were performed using the igraph and ggraph packages in R.

Differential Gene Expression Analysis

Filtering and Feature Selection

Prior to differential expression analysis, low-count features were filtered to improve statistical power and reduce multiple testing burden. Canonical genes were retained if they had at least 10 counts in a minimum of 5 samples, while transposable elements (LINE1s and HERVs) were filtered using lower thresholds of 5 counts in at least 3 samples due to their typically lower expression levels. This filtering strategy reduced the feature set to 34,335 tested features, retaining 21,622 canonical genes 8,438 LINE1 elements, and 4,275 HERV elements.

Statistical Analysis

Differential expression analysis was performed using DESeq2 (10) with a design formula of *~phenotype* to compare gene expression between schizophrenia patients and healthy controls. The DESeq2 pipeline included median-of-ratios normalization for library size differences, dispersion estimation, and negative binomial generalized linear model fitting. Results were extracted using the contrast "schizophrenia" vs "control" to identify features with significantly altered expression. Multiple testing correction was performed using the Benjamini-Hochberg method, with statistical significance defined as adjusted p-value (padj) < 0.01.

A total of 14,265 features showed significant differential expression (padj < 0.01) out of 34,335 tested features (41.5%), with 5,583 upregulated and 8,682 downregulated in schizophrenia compared to controls. The differential expression pattern varied substantially across feature types: canonical genes showed the highest proportion of significant changes with 11,531 out of 21,622 genes (53.3%) differentially expressed, including 5,241 upregulated and 6,290 downregulated. In contrast, transposable elements showed lower but notable differential expression rates, with 1,745 out of 8,438 LINE1 elements (20.7%) and 989 out of 4,275 HERV elements (23.1%) significantly altered. Both LINE1 and HERV elements showed predominantly downregulated expression in schizophrenia, with

1,565 of 1,745 significant LINE1 elements (89.7%) and 827 of 989 significant HERV elements (83.6%) showing decreased expression. The most extreme expression changes were observed in LINE1 elements, with *L1FLnI_7p21.1n* showing the strongest downregulation (log2FC = -3.80, padj = 2.23×10^{-8}), while the canonical gene GJB6 exhibited the most significant upregulation (log2FC = 3.56, padj = 8.77×10^{-61}) (Supplementary Figure 1F-G).

Differential Gene Expression Visualization Methods

Volcano Plots

Differential expression results were visualized using volcano plots (11) displaying log2 fold change versus -log10(adjusted p-value) for all tested features. Points were colored by biotype (canonical genes, LINE1 elements, HERV elements) and sized according to statistical significance. Significance thresholds were indicated by dashed lines at padj = 0.01 (horizontal) and |log2FC| = 0.5 (vertical). The most significant genes from each category and top differentially expressed features were labeled.

Heatmaps

Expression patterns of the most significant features were visualized using hierarchical clustering heatmaps (12). Variance-stabilizing transformation (VST) was applied to normalized counts, followed by Z-score normalization across samples for each gene to enable comparison of relative expression changes. The top 50 features were selected based on a combined score incorporating both statistical significance and effect size (-log10(padj) × |log2FoldChange|). Heatmaps were generated with Euclidean distance and complete linkage clustering for both rows (genes) and columns

(samples). Sample phenotypes and gene types were annotated using color bars, with samples clustered to reveal expression patterns associated with disease status.

Effect Size Analysis

Composition Analysis by Log2 Fold Change Thresholds

To characterize the relationship between effect size and gene type composition among differentially expressed features, we performed a systematic analysis of significant genes (padj < 0.01) across increasing log2 fold change thresholds. Six thresholds were evaluated: 0, 0.25, 0.5, 0.75, 1.0, and 1.5, corresponding to no effect size filter, 1.19-fold, 1.41-fold, 1.68-fold, 2-fold, and 2.83-fold changes, respectively. For each threshold, we calculated the total number of genes meeting the criteria, the percentage of all significant genes retained, and the composition breakdown by biotype (Supplementary figure 1C-E).

This analysis revealed a compositional shift as effect size requirements increased, with TEs progressively dominating larger expression changes. While canonical genes comprised 80.8% of all significant changes (no effect size filter), their representation decreased to only 19.5% among the most extreme changes ($|\log2FC| \geq 1.5$). Conversely, L1 elements showed the most pronounced compositional shift, increasing from 12.2% to 62.9% of high-effect changes. HERV elements maintained relatively consistent representation of 15-17% across higher thresholds, indicating their persistent contribution to large expression changes.

Genome-wide Suppression of Repetitive Elements

Features with the most extreme expression changes (|log2FC| ≥ 1.5 and padj < 0.01) revealed near-universal suppression of TE expression in schizophrenia. Among 931 features showing extreme dysregulation, canonical genes represented only 19.5% (182 genes), with LINE1 elements accounting for 62.9% (586 genes) and HERV elements representing 17.5% (163 genes) of extreme changes, demonstrating that the largest transcriptional perturbations in schizophrenia affect TEs.

The directional pattern of dysregulation was consistent: 99.3% of extreme LINE1 changes (582/586) and 98.2% of extreme HERV changes (160/163) showed decreased expression in schizophrenia. The most extreme individual changes exemplified this pattern, with the LINE1 element *L1FLnI_7p21*.1n showing the strongest downregulation (log2FC = -3.8, 14-fold decrease) while the canonical gene GJB6 exhibited the most significant upregulation (log2FC = +3.56, 11.8-fold increase).

Assessment of demographic confounding

The SCZ and HC groups differ in sex composition (SCZ: 48.8% male, n = 41/84; HC: 29.9% male, n = 29/97) and mean age (SCZ: 27.9 ± 7.3 years; HC: 20.9 ± 2.3 years). To quantify the maximum possible confounding from the sex composition difference, we calculated an upper bound on the apparent log2 fold change that the 19 percentage point sex imbalance could produce.

Differential Expression. DESeq2 compares average expression between the SCZ and HC groups. Each group's average is the sum of its individual sample values divided by the group size. If the TE expression were entirely determined by sex (every male at expression level "L" and every female at expression level "H"), then each group's average would simply be the weighted average of male and female expression, with the weights set by the sex composition of that group:

$$\text{HC mean} = (29/97) \times L + (68/97) \times H = 0.299L + 0.701H$$

SCZ mean = (41/84) × L + (43/84) × H = 0.488L + 0.512H

The apparent log2 fold change would be log2(SCZ mean / HC mean). Even in the most extreme case where one sex has zero expression (L=0), this yields:

|log2FC| = 0.45

This is 3.3 times smaller than the |log2FC| ≥ 1.5 threshold used to define extreme expression changes and 8.4 times smaller than the largest observed L1 effect (log2FC = -3.8 for *L1FLnI_7p21.1n*). In reality, expression is not entirely determined by sex and individual variation exists, so the actual confounding would be smaller. Therefore, the sex composition difference is mathematically insufficient to produce the observed effect sizes.

The same reasoning applies to the 7 year mean age difference (except instead of H and L we would look at a continuous variable of rate of change for year) with even smaller expected confounding effect.

Mutual Information networks. The HC and SCZ networks were constructed independently, each capturing coexpression patterns across individuals within one condition. The MI between two genes reflects whether their expression levels covary across individuals, not their mean expression levels. All 84 SCZ samples share a diagnosis of first-episode treatment-naive schizophrenia. The coexpression relationships that emerge with statistical regularity across the mixed-sex, mixed-age cohort represent the shared biology of the condition. Demographic heterogeneity within a group adds noise that attenuates MI estimates and makes edges harder to detect; it does not create systematic coexpression between hundreds of TEs and thousands of canonical genes. Both the HC and SCZ groups contain males and females, so any sex-driven coexpression structure would appear in both networks and would not explain differences between them.

Future studies with covariate data at the individual level should formally evaluate the contribution of sex and age to TE-gene PBMCs coexpression networks in schizophrenia.

Study Approval:

No human subject approval was needed. The RNA-seq data deposited in GEO GSE243841 is publicly available and deidentified.

Data Availability:

This study analyzed publicly available RNA-seq data deposited in the Gene Expression Omnibus (GEO: GSE243841) (1).

See also supporting data values file.

Supporting analytic code for the manuscript can be accessed at: https://github.com/hreypar/bulk_scz_pbmc

Acknowledgments:

The views expressed are those of the authors and not necessarily those of the NHS, the NIHR, or the Department of Health and Social Care.

Author Contributions

HRG and DFN conceived the study. HRG, MLB, RRRD, TRP and DFN performed the experiments for the study. HRG and DFN wrote the manuscript. All authors revised the manuscript.

References:

1. Pan B, et al. Identifying periphery biomarkers of first-episode drug-naïve patients with schizophrenia using machine-learning-based strategies. *Prog Neuropsychopharmacol Biol Psychiatry*. 2025;137(111302):111302.

2. Frankish A, et al. GENCODE reference annotation for the human and mouse genomes. *Nucleic Acids Res*. 2019;47(D1):D766–D773.

3. Ho JSY, Douse CH, Marazzi I. Transposable element DNA and RNA: Drivers of gene expression, evolution, and disease. *Cell*. 2026;189(12):3513–3540.

4. Dobin A, et al. STAR: ultrafast universal RNA-seq aligner. *Bioinformatics*. 2013;29(1):15–21.

5. Bendall ML, et al. Telescope: Characterization of the retrotranscriptome by accurate estimation of transposable element expression. *PLoS Comput Biol*. 2019;15(9):e1006453.

6. Malod-Dognin N, et al. Chromatin network markers of leukemia. *Bioinformatics*. 2020;36(Suppl_1):i455–i463.

7. Margolin AA, et al. ARACNE: an algorithm for the reconstruction of gene regulatory networks in a mammalian cellular context. *BMC Bioinformatics*. 2006;7 Suppl 1(S1):S7.

8. Meyer PE, Lafitte F, Bontempi G. minet: A R/Bioconductor package for inferring large transcriptional networks using mutual information. *BMC Bioinformatics*. 2008;9(1):461.

9. Csárdi G, et al. igraph: Network Analysis and Visualization in R [preprint]. 2026. https://doi.org/10.5281/zenodo.7682609.

10. Love MI, Huber W, Anders S. Moderated estimation of fold change and dispersion for RNA-seq data with DESeq2. *Genome Biol*. 2014;15(12):550.

11. Blighe K. *EnhancedVolcano*. Bioconductor; 2018.

12. Kolde R. pheatmap: Pretty Heatmaps [preprint]. 2025. https://doi.org/10.32614/CRAN.package.pheatmap.

Supplementary figure legends

Supplementary Figure1. (A) Topological properties of SCZ and HC mutual information networks. (B) Gene detection rate by feature (transcript) type. (C) Number of transcripts with extreme expression changes in SCZ. (D) Effect size distribution for transcripts with extreme expression changes. (E) Top 20 differentially expressed genes, L1s, and HERVs. (F) Differentially expressed TE transcripts that distinguish PBMCs of SCZ from HC. (G) GJB2 and GJB6 expression in SCZ and HC samples. GC (canonical genes). L1 (LINE1).

Supplementary Figure 2. Properties of the mutual information edges in the SCZ and HC networks. (A) Mutual Information values distribution in SCZ and HC. (B) Average MI edge value between different biotypes. (C) Number of MI edges between different biotypes. TE (Transposable Element). TF (Transcription Factor). MI (Mutual Information). SCZ (Schizophrenia). HC (Healthy controls).

Supplementary Figure 3. (A) Dotplot of pathways enriched for genes that exhibit high connectivity to TE elements (HERV and L1) and are disrupted in SCZ.

Supplementary Figure 4. (A) Degree distribution of genes (blue) and TEs (red). (B) Number of TE hub nodes by TE type. (C) Network graphs of TEs rewired in SCZ compared to HC. LINE1 (LINE1s).

A

	SCZ Network	HC Network
Nodes	16,041	16,041
Edges	1,725,564	3,538,017
Density	0.0134	0.0275
Average Degree	215.1	441.1
Clustering Coefficient	0.139	0.188

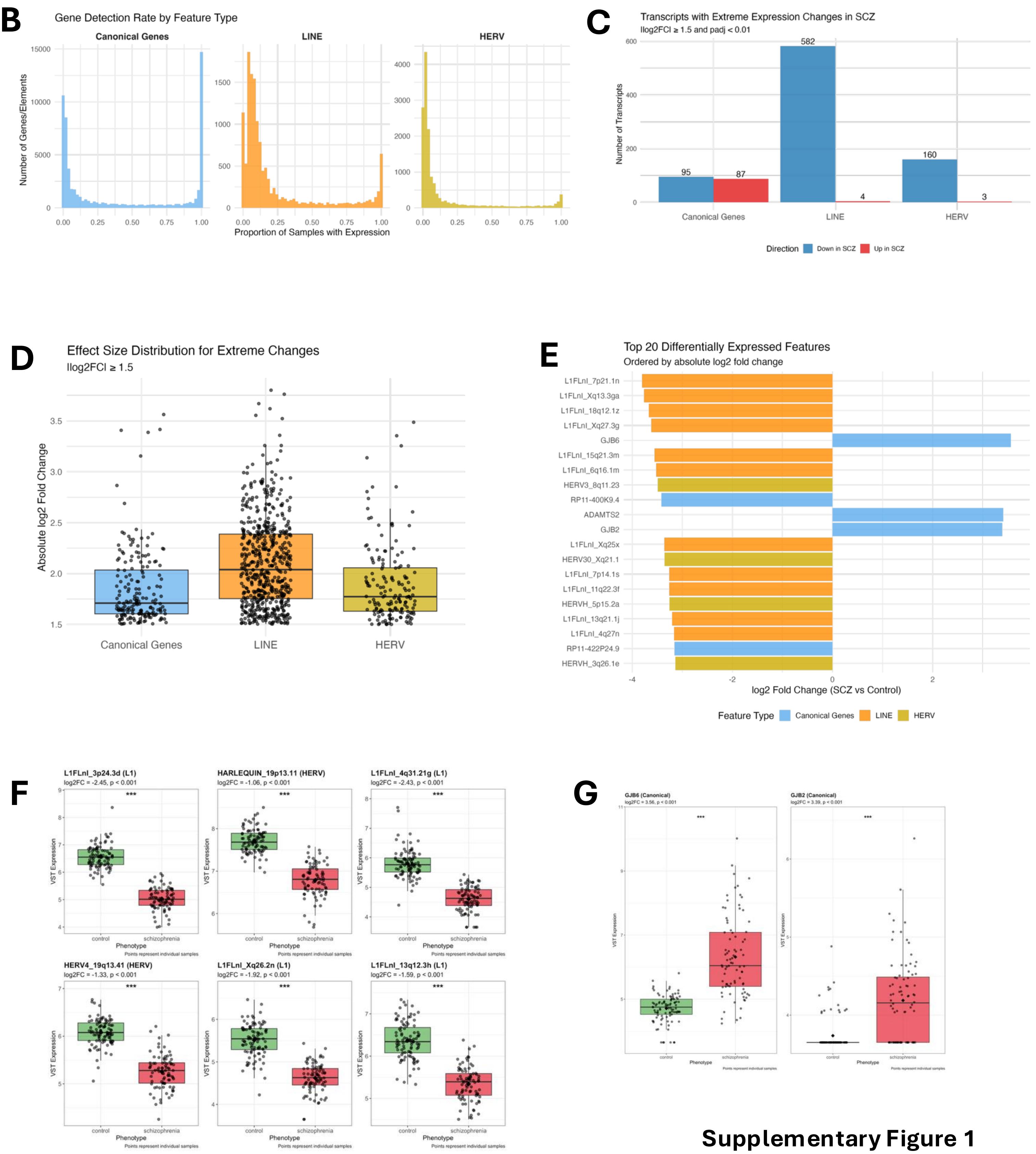

Supplementary Figure 1

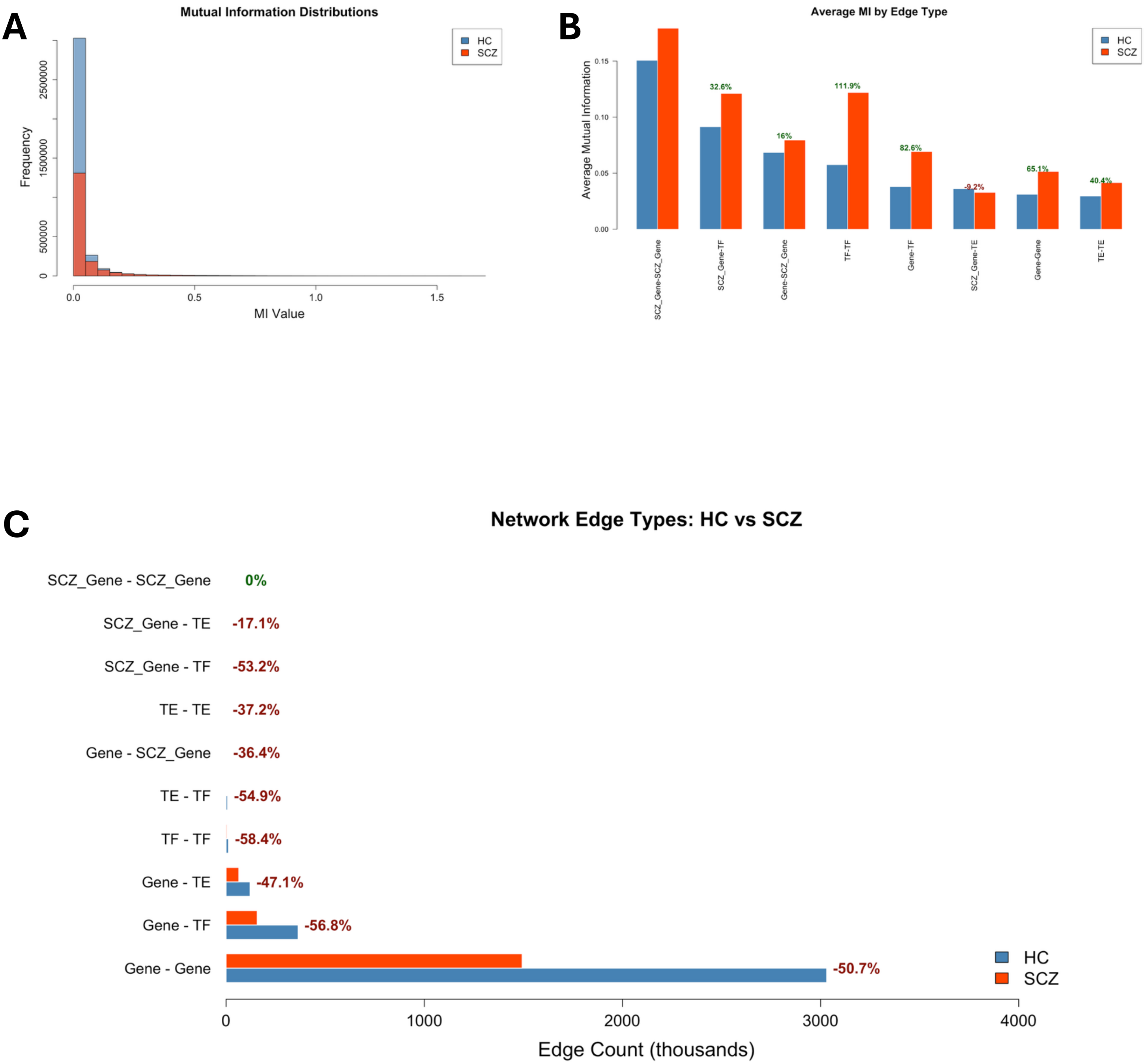

Supplementary Figure 2

A

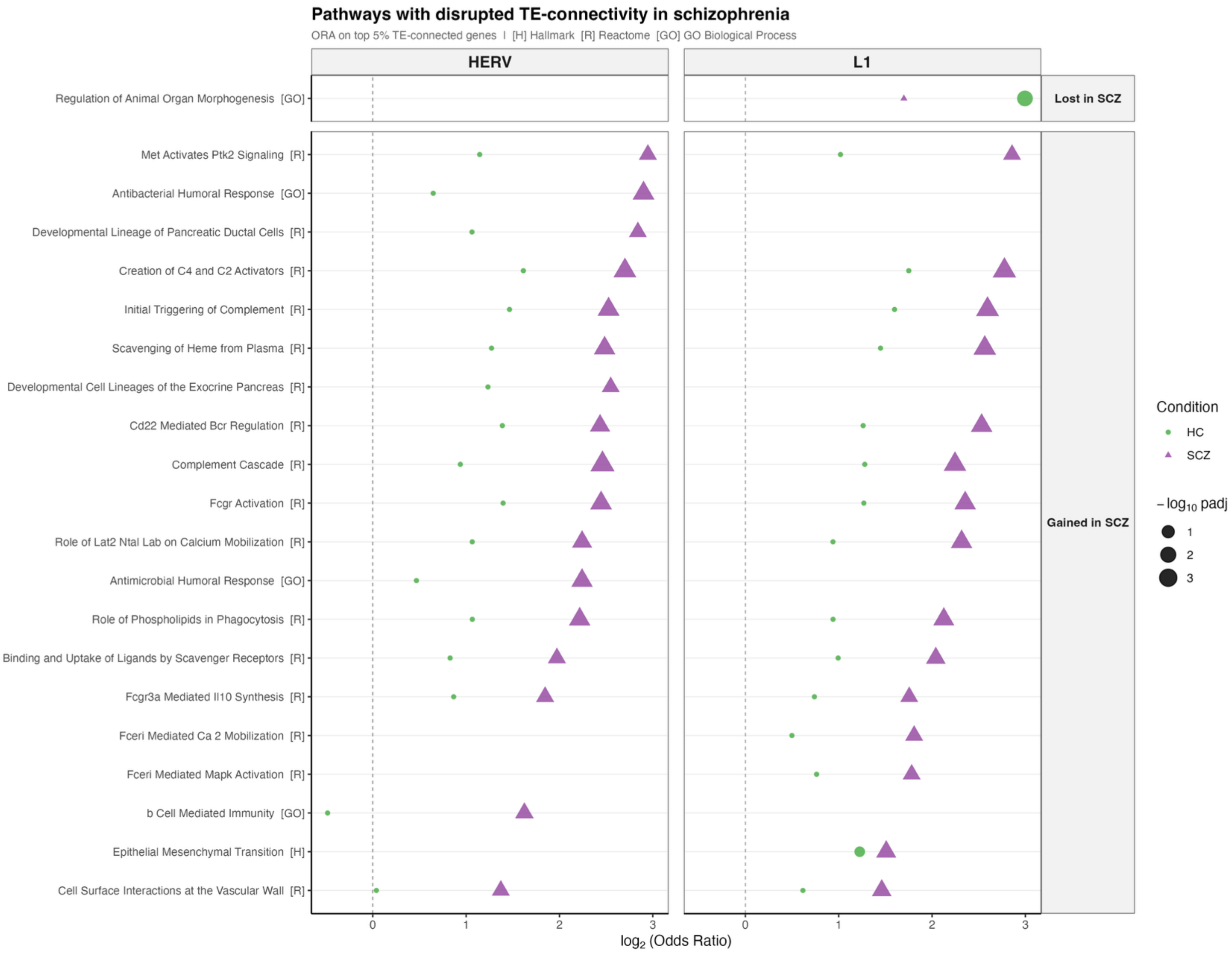

Supplementary Figure 3

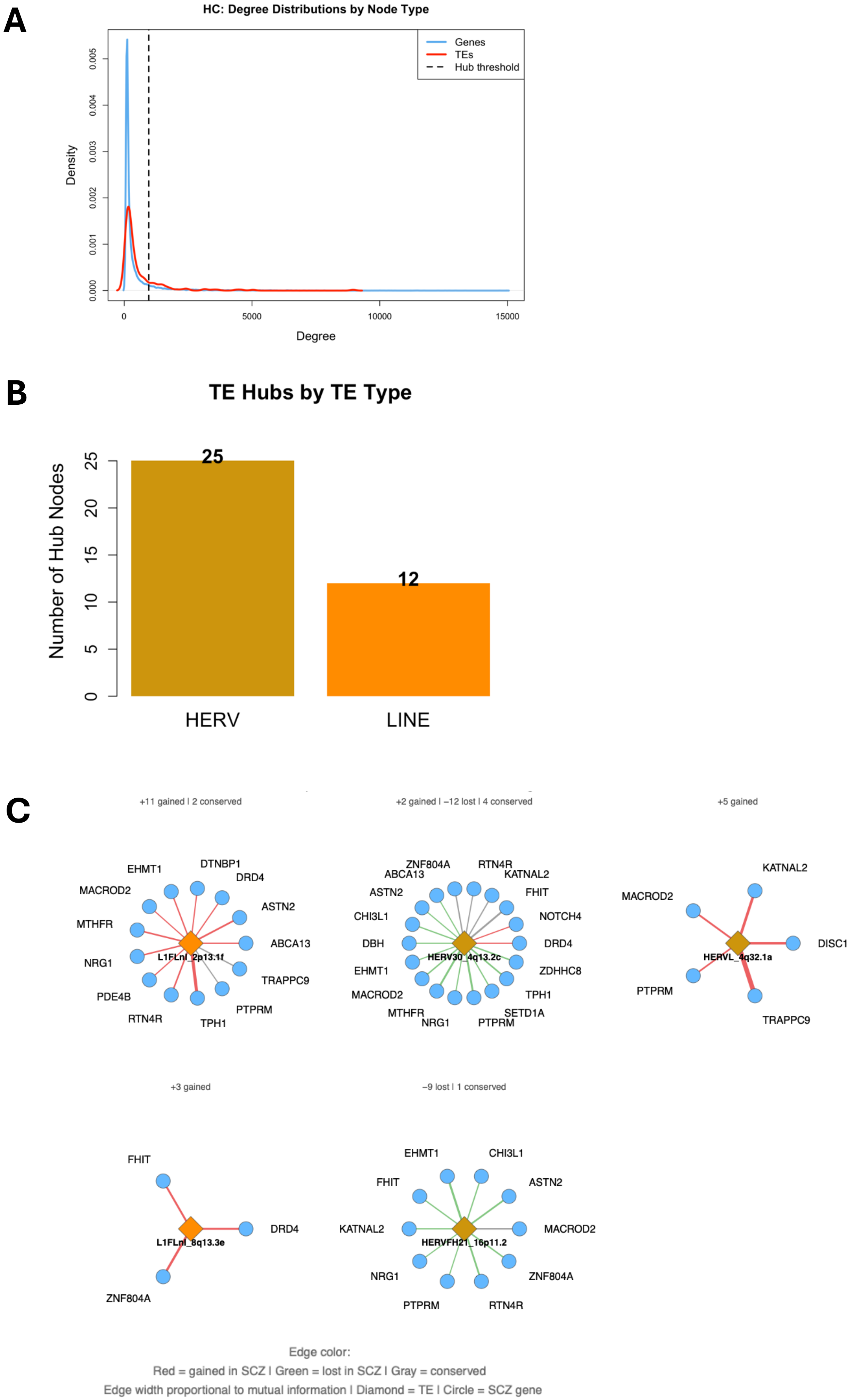

Supplementary Figure 4
